# Estimating transmission probability in schools for the 2009 H1N1 influenza pandemic in Italy

**DOI:** 10.1186/s12976-016-0045-2

**Published:** 2016-10-12

**Authors:** Valentina Clamer, Ilaria Dorigatti, Laura Fumanelli, Caterina Rizzo, Andrea Pugliese

**Affiliations:** 1 Department of Mathematics, University of Trento, Via Sommarive 14, Trento, 38123 Italy; 2 MRC Centre for Outbreak Analysis & Modelling, Department of Infectious Disease Epidemiology, Imperial College, London, UK; 3 Center for Information Technology, Bruno Kessler Foundation, Trento, Italy; 4 Istituto Superiore di Sanità, Roma, Italy

**Keywords:** Influenza, Transmission probability in schools, Bayesian inference, Discrete-time SIR epidemic model

## Abstract

**Background:**

Epidemic models are being extensively used to understand the main pathways of spread of infectious diseases, and thus to assess control methods. Schools are well known to represent hot spots for epidemic spread; hence, understanding typical patterns of infection transmission within schools is crucial for designing adequate control strategies. The attention that was given to the 2009 A/H1N1pdm09 flu pandemic has made it possible to collect detailed data on the occurrence of influenza-like illness (ILI) symptoms in two primary schools of Trento, Italy.

**Results:**

The data collected in the two schools were used to calibrate a discrete-time SIR model, which was designed to estimate the probabilities of influenza transmission within the classes, grades and schools using Markov Chain Monte Carlo (MCMC) methods. We found that the virus was mainly transmitted within class, with lower levels of transmission between students in the same grade and even lower, though not significantly so, among different grades within the schools. We estimated median values of *R*_0_ from the epidemic curves in the two schools of 1.16 and 1.40; on the other hand, we estimated the average number of students infected by the first school case to be 0.85 and 1.09 in the two schools.

**Conclusions:**

The discrepancy between the values of *R*_0_ estimated from the epidemic curve or from the within-school transmission probabilities suggests that household and community transmission played an important role in sustaining the school epidemics. The high probability of infection between students in the same class confirms that targeting within-class transmission is key to controlling the spread of influenza in school settings and, as a consequence, in the general population.

**Electronic supplementary material:**

The online version of this article (doi:10.1186/s12976-016-0045-2) contains supplementary material, which is available to authorized users.

## Background

Epidemic models are being extensively used to understand the main pathways of spread of infectious diseases, and thus to assess control methods. Generally, they are fitted to rather aggregated datasets reporting the number of new cases (possibly stratified by age or other variables of interest) in each time interval (often a week, although sometimes daily reports are available, especially at the initial outbreak of an infection). In some cases, data on all individuals of a small community have been available [1], and this has allowed obtaining a better understanding of the person-to-person spread. Still, the question arises of whether small isolated communities are representative of disease spread in more usual contexts. The attention that was given to the 2009 A/H1N1pdm09 influenza pandemic has made it possible to collect detailed data on the epidemic spread in more typical contexts. Schools are well known to represent hot spots for epidemic spread [2–11]. Contact rates within schools are generally higher than outside, as was noticed in [3, 12, 13]. Using detailed data on an outbreak of 2009 pandemic influenza in a school, Cauchemez et al. [14] estimated the different infection probabilities within each class, or grade, and in the whole school, as well as quantified the spread through other household members, and were also able to assess the role of heterogeneities in contact rates. In this work we provide estimates for transmission rates of 2009 A/H1N1pdm09 pandemic influenza at the three levels of class, grade and school by analyzing data on the occurrence of influenza-like illness (ILI) symptoms among pupils of two primary schools in Trento (Italy). The data were collected retrospectively in December 2009, a few weeks after the epidemic peak, through a questionnaire delivered to the parents of the pupils attending the two primary schools. The overall vaccination rate in the age-group was extremely low in Italy (0.3 %) and the use of antiviral drugs was recommended by the Italian Ministry of Health only for severe cases of pandemic influenza and for symptomatic patients with underlying medical conditions [15]. Although we did not ask specific information about responses to influenza, it seems unlikely that antiviral or vaccine use have significantly affected the epidemic dynamics in the two schools. We developed a discrete-time SIR model to analyze the collected data, where the transmission parameters were then estimated via Markov chain Monte Carlo methods, appropriate to make parameter inference in presence of missing data [16, 17]. In order to understand the power of the method, we also applied the algorithm to simulated data, generated to reproduce a school structure, under several hypotheses on the transmission dynamics. This work on simulated data made us, on the one hand, get a better interpretation of the results obtained, showing for instance to which degree parameters are identifiable; on the other hand, assess the loss in accuracy resulting from missing data and other sources of error.

## Methods

### Data

**Fig. 1:**
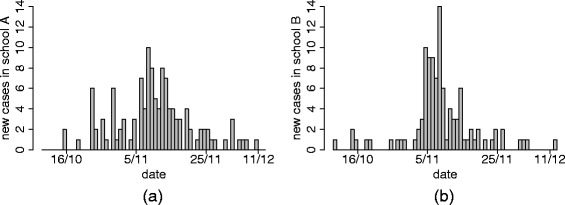
Incidence curves in the two schools. **a** Daily number of new cases in school A. **b** Daily number of new cases in school B

In December 2009 we delivered a questionnaire to the parents of the pupils of two primary schools in Povo (school A) and Villazzano (school B), two suburbs of Trento (Italy). School A consisted of 307 students divided into 14 classes of 5 different grades, while school B consisted of 214 students divided into 10 classes of 5 different grades. As far as we know, no significant changes occurred in the school composition during the study period (October-December 2009). The questionnaire (see Section A in the Additional file 1 for an abridged English translation) reported a description of ILI symptoms, asked the parents to report whether any member of the family had experienced ILI symptoms in the preceding months and, if that was the case, to report the date of symptoms onset (or an estimate of it) for each member of the family, similarly to what was done in [3, 4]. Table 1 and Fig. 1 describe the data collected in the two primary schools. Complete data are available in Additional files 2 and 3. The information provided on all the other members of the families were scarce and for this reason they were excluded from this study.

**Table 1:** Summary of the main features emerging from the questionnaires collected in schools A and B in Trento, Italy in 2009

	School A	School B
School size	307	214
Number of classes	14	10
Number of responses	260	168
Number of ILI cases	121	103
Response rate	85 %	79 %
Reported Attack rate	46 %	61 %

### Epidemic model and parameter estimation

The epidemic process is described using a discrete-time SIR model, with a time step of 1 day. Following [18], we assume that the incubation period (time from infection to symptom occurrence) is on average 2 days and varies between 1 and 3 days, and that the infectiousness profile is as described in [19]. Furthermore, we assume that, after the development of ILI symptoms, a child is kept at home, according to the usual practice in Italy. Combining this assumption with the estimate of the incubation period, we conclude that if a child is infected at school on day *t*, he/she will be at school and infectious on day *t*+1; on day *t*+2 he/she will be infectious and either kept at home, or still at school with probability *γ* (we estimate *γ*=0.1 from Figures 1b) and 1d) of [18]); after that, she/he will certainly be kept home and will not contribute further to within-school transmission. The assumptions are consistent with the estimate in [14] of 1.1 days for the within-school generation time. Hence, we assume that the school population can be divided into: susceptible individuals *S*, infectious individuals *I* (infected children who can transmit the disease, divided into two sub-compartments *I*_1_ and *I*_2_ depending on them being in the first or second day of infectiousness, respectively) and recovered individuals *R* (including both recovered children and children kept at home after symptoms onset).

The model is a Markov chain where the transitions are given by

$$S \to I_{1},\qquad I_{1} \mathop{\to}^{\gamma} I_{2} \qquad I_{1} \mathop{\to}^{1-\gamma} R \qquad I_{2} \mathop{\to}^{1} R. $$

Each susceptible individual may become infected (transition *S*→*I*_1_) upon contacts with infected individuals. For the sake of simplicity, we assume that individuals in compartments *I*_1_ and *I*_2_ are equally infectious. Furthermore, following [14], we assume different probabilities of infection by setting: within-class (*q*_*c*_), in the same grade but in a different class (*q*_*g*_), in the same school but in a different grade (*q*_*s*_) and in households or in the general community (*ε*). We define

- $I_{t}^{{j,h}}$ the number of infectious students (either in their first or second day of infectiousness) in grade *j*, class *h* at time *t*;
- $I_{t'}^{{j,h}} = \sum _{k\not = h} I_{t}^{{j,k}} $ the number of infectious students in the classes of grade *j* other than *h* at time *t* $= {I_{t}^{j}} $ (number of infectious students in all classes of grade *j*) $ - I_{t}^{{j,h}}$ ;
- $I_{t^{\prime \prime }}^{j} = \sum _{i \not = {j, h}} I_{t}^{{i,h}}$ the number of infectious students in grades other than *j* at time *t* =*I*_*t*_ (number of infectious individuals in all classes of the school) $ - {I_{t}^{j}}$,

The probability for a susceptible student in grade *j*, class *h* to remain susceptible is

1 $$  p_{t}^{j,h} = (1 - q_{c})^{I_{t}^{j,h}} (1 - q_{g})^{I_{t'}^{j,h}}(1 - q_{s})^{I_{t^{\prime\prime}}^{j}} (1-\varepsilon)  $$

and $1 - p_{t}^{{j,h}} $ is the probability of becoming infectious and moving into compartment *I*_1_ at time *t*+1.

Then the probability of having $S_{t+1}^{{j,h}}$ susceptibles at time *t*+1, by considering the school at time *t*, can be obtained as

2 $$ P\left(S_{t+1}^{j,h}|S_{t}^{j,h},I_{t}^{j,h},{I_{t}^{j}},I_{t}\right)=\binom{S_{t}^{j,h}}{S_{t+1}^{j,h}}\left({p_{t}^{j,h}}\right)^{S_{t+1}^{j,h}} \left(1-p_{t}^{j,h}\right)^{\left(S_{t}^{j,h}-S_{t+1}^{j,h}\right)}  $$

The full list of variables and parameters of the model is reported in Table 2. Model parameters have been estimated using the MCMC Metropolis-Hastings method, as described in [16, 20], and the code was written in *C*. The estimated parameters are the infection probabilities within class *q*_*c*_, within the same grade *q*_*g*_, among different grades of the schools *q*_*s*_ and from outside the schools *ε*; we thus refer to this model as model CGS (Class-Grade-School). The augmented data are all unobserved events such as the infection dates and the infection state of the children whose questionnaires were not filled.

**Table 2:** Model parameters and variables

Symbol	Description
*q*_*c*_	Within-class infection probability
*q*_*g*_	Same grade infection probability
*q*_*s*_	Within-school infection probability
*ε*	Outside-school infection probability
*γ*	Probability to remain infective for two days
*I*_*t*_	Number of infective subjects at time *t* in the whole school
${I_{t}^{j}}$	Number of infective subjects at time *t* in grade *j*
$I_{t}^{j,h}$	Number of infective subjects at time *t* in grade *j* and class *h*
$S_{t}^{j,h}$	Number of susceptible individuals at time *t* of grade *j* and class *h*
*n*_*j*_	Number of classes of grade *j*

### Model variants

We considered the following two simplifications: model CS (Class-School) where we differentiate between within-class transmission and within-school transmission only, without considering a separate probability of transmission within the grade; and model S (School only), where we assume that the probability of transmission is the same for all students in the school. We explore a further variant of model CGS (CGS-var), where the probability of infection from outside the school, instead of being constant over time, is assumed to be proportional (through a constant *ε*) to the ILI incidence in the corresponding week in the province of Trento, as reported by the surveillance system InfluNet of the Italian Institute of Health [21].

We compare the model variants using an adapted version of the deviance information criterion (DIC) described in [22, 23]. Specifically, distinguishing between actual model parameters (*θ*) and unobserved events (*Y*), we computed a marginalized DIC as

$$ \text{DIC} = - 4 \mathbb{E}_{(\theta,Y)}\log\left(L(X,Y|\theta)\right) + 2 \mathbb{E}_{Y} \log\left(L(X,Y|\bar \theta)\right) $$

where *X* are the observed data, while *L*(·|*θ*) is the likelihood of the complete data under the Markov chain with parameters *θ*.

### Tests on simulated data

We tested the model and the estimation algorithm on simulated data obtained using model CGS under different parameterizations (see the Additional file 1 for details).

### Reproduction number

**Fig. 2:**
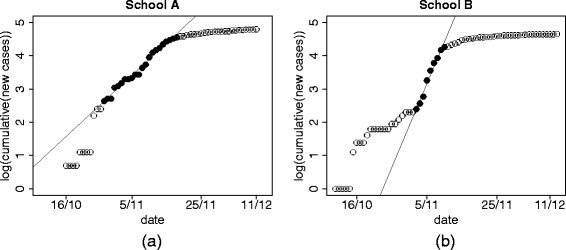
Estimation of exponential growth rates. Cumulative infection data (in log-scale) for school *A* (panel **a**) and school *B* (panel **b**). Black points were used in the linear regression procedure for estimating the epidemic growth rate

A typical summary indicator of an epidemic is its basic reproduction number *R*_0_, which represents the expected number of secondary cases generated by a single typical infection in a completely naive population. As we do not have data on actual infections but only on the occurrence of symptoms, we can estimate what we may consider a school-specific effective reproduction number. *R*_0_ can be estimated through the rate of initial epidemic growth *r* using the formula *R*_0_=1+*r* *T*_*I*_ [24], where *T*_*I*_ represents the mean generation time; *r* has been estimated through the fit of a linear model either to the incidence data (grouped by 3 days) or to the cumulative number of cases (see [25] for a statistical analysis of the consequences of either choice) in the log-scale (Fig. 2 shows the fit to the curve of cumulative cases over a specific time window).

The classical definition of *R*_0_ in a finite population stochastic model is generally based on the limit as the population grows to infinity (see, for instance, [26]). Instead of doing this, we rely on a simple operational definition, namely we define *R*_0_ as the average number of students infected by the first infected student in the school. By assuming to have *n*_*s*_ grades (5 in Italian primary schools), each with *n*_*g*_ classes with *n* students, then we obtain

3 $$  R_{0} = (q_{c} (n-1) + q_{g} n (n_{g}-1) + q_{s} n n_{g} (n_{s} - 1))(1+\gamma).  $$

## Results

The overall response rate to the questionnaire was 82 *%*(428/521) and the reported ILI cases were 224 (52 *%*) (see Table 1). In school A, the first two cases were reported on 16 October 2009 and the last case was reported 56 days later; in school B, the first case was reported on 10 October 2009 and the last case occurred 64 days later (Fig. 1).

We estimated the initial growth rate *r* both from the (grouped) incidence curve and from the cumulative curve (Fig. 2), selecting those time windows in the growing part of the epidemic for which *R* ^2^ was sufficiently high (>0.95 for the cumulative curve, >0.7 for the incidence curve); assuming that the infectious period at school *T*_*I*_ is 1.1 day, we obtained a median *R*_0_ of 1.16 for school A and 1.40 for school B; the overall range of confidence intervals (obtained from the different time windows) is 0.93-1.43 for school A; 1.08-1.76 for school B using the fit from incidence curves. The intervals obtained from cumulative curve are much narrower, but may be deceivingly so [25].

**Fig. 3:**
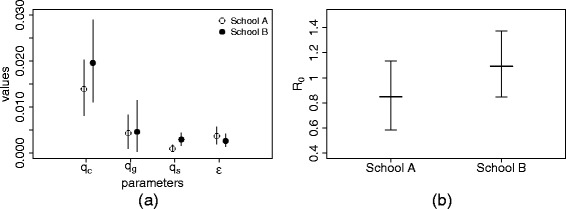
Estimated values of the transmission parameters and of *R*_0_. **a** Estimated values of the transmission parameters for school A and B. White and black dots represent the mean of the posterior distribution for school A and school B respectively, bars represent 95 *%*-credible intervals. **b** Estimated values of the reproduction number *R*_0_ inside schools A and B. Thick line and bars represent means and 95 *%*-credible intervals

Table 3 summarizes the estimated infection probabilities within class *q*_*c*_, in the same grade except the class *q*_*g*_, within the school except the grade *q*_*s*_ and from outside the school for schools A and B; Fig. 3 a presents a comparison of the estimates obtained for the two schools. The most evident feature of these results is that, for both schools, the estimated class infection transmission probability is the highest of all settings. Grade transmission probability is estimated in both schools to be higher than school transmission; however the respective 95 %-credible intervals overlap (just barely in school A, largely in school B).

**Table 3:** Transmission probabilities estimates

Parameters	School A	School B
*q*_*c*_, mean [ 95 *%* CI]	1.39×10^−2^ [ 8.10×10^−3^−2.03×10^−2^]	1.96×10^−2^ [ 1.11×10^−2^−2.89×10^−2^]
*q*_*g*_, mean [ 95 *%* CI]	4.36×10^−3^ [ 9.61×10^−4^−8.34×10^−3^]	4.61×10^−3^ [ 2.98×10^−4^−1.15×10^−2^]
*q*_*s*_, mean [ 95 *%* CI]	9.52×10^−4^ [ 2.87×10^−4^−1.82×10^−3^]	2.96×10^−3^ [ 1.64×10^−3^−4.45×10^−3^]
*ε*, mean [ 95 *%* CI]	3.70×10^−3^ [ 1.95×10^−3^−5.69×10^−3^]	2.65×10^−3^ [ 1.37×10^−3^−4.20×10^−3^]

Mean and 95 *%*-credible intervals of the estimates for the infection probabilities in schools A and B, when considering model CGS

As for comparisons between the two schools, estimates of class and grade transmission probability are similar, as is the probability of transmission from outside the school. On the other hand, estimates of transmission probability within school are rather different (95 %-credible intervals barely overlap).

Using these estimates for transmission probabilities, we obtain from Eq. (3) the values of *R*_0_ shown in Fig. 3 b, with an average of 0.85 in school A and 1.09 in school B. Note that Eq. (3) is based only on within-school transmission and does not include transmissions to household members or acquaintances; on the other hand, the estimates based on Fig. 2 depend on all infected students, whatever their source of infection.

DIC values for the four different models considered in this study (see “Model variants” section) are presented in Table 4. For school A, model CGS is clearly to be preferred to the others because its DIC value is much lower. For school B, model CGS with constant *ε* is, according to DIC value, only slightly better than model CS, and both are definitely to be preferred to model S; on the other hand, model CGSvar outperforms all the others.

**Table 4:** Model comparison

Model	School A	School B
**CGS** (*q*_*c*_,*q*_*g*_,*q*_*s*_,*ε*)	702.83	757.91
**S** (*q*_*c*_=*q*_*g*_=*q*_*s*_,*ε*)	799.91	779.38
**CS** (*q*_*c*_,*q*_*g*_=*q*_*s*_,*ε*)	774.91	761.16
**CGSvar** (*q*_*c*_,*q*_*g*_,*q*_*s*_,*ε*_*var*_)	751.20	426.21

DIC values of the different models considered. Model CGS has three different transmission rates inside the school (*q*_*c*_, *q*_*g*_ and *q*_*s*_). Model S has a homogeneous infection rate inside the school (*q*_*c*_). Model CS has a transmission rate for the class (*q*_*c*_) and a different transmission rate in the remaining part of the school (*q*_*g*_). Model CGSvar is the same as CGS but with a non-constant *ε*

**Fig. 4:**
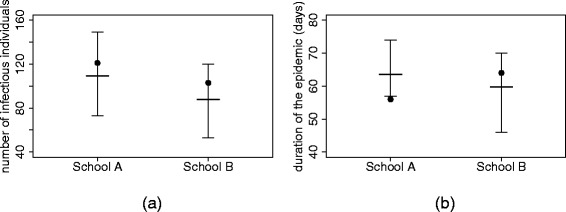
Comparison between observed data and simulations. Plot of the total number of infectious individuals (panel **a**) and the duration of the epidemic (panel **b**) in 400 simulations. The black dot indicates the observed number of infectious individuals and the observed length of the epidemic in the two schools. Thick line and bars represent means and 95 *%*-credible intervals

In order to assess whether the CGS model is compatible with the data, we performed 400 simulations (for each school) having randomly drawn the parameter values from the corresponding posterior distributions. The model was then compared with the data (see Fig. 4) through two different indicators: the total number of infected children and the total length of the epidemic.

Finally, we tested the model and the estimation algorithm on simulated data obtained using model CGS under different parameterizations and found that the infection probabilities *q*_*c*_, *q*_*g*_, *q*_*s*_ and *ε* were successfully identified (see Table and Figure S2 in the Additional file 1).

## Discussion

We estimated influenza transmission probabilities in a school setting, using the data collected through a retrospective survey conducted in December 2009 in two primary schools and we found that, in both schools, influenza was mainly transmitted within classes (Fig. 3). Same- and different-grade transmission, as well as outside-school transmission, were all significantly lower than within-class transmission, with no significant difference between them (Fig. 3).

We found that for both primary schools model CGS (that distinguishes within-class, same-grade and different-grade transmission) has the lowest DIC, i.e. is the favorite model overall. According to the DIC, models CGS and CS (that distinguishes within-class transmission from the general within-school transmission only) are equivalently good for school B, which reflects the similarity observed in the estimated same-grade and different-grade transmissions (Fig. 3).

Similar results were obtained in [14], where the transmission probability between students of the same class was estimated to be five times larger than the transmission probability between students of the same grade and, in turn, this was five times larger than the transmission probability between students of different grades. The estimates we obtained are similar, with factors of 3-4 instead of 5, except for the grade/school ratio in school B, which is just above 1. These results are also consistent with the studies presented in [27, 28], where wearable sensors were used to determine the structure of contacts in schools: these studies found that children spent on average three times more time with children of the same class than with children of other classes. The fact that within-class transmission is estimated to be higher than within-school transmission can have implications on the design of school closure policies aimed at mitigating the spread of influenza, especially on evaluating the effectiveness of gradual closures (where single classes close first, then grades and finally the entire school) [27, 29–31].

Another interesting result emerges from the comparison between the two schools involved in the study: while the estimates for within-class *q*_*c*_ and within-grade *q*_*g*_ transmission probabilites are similar for the two schools, the estimate for school-wide transmission *q*_*s*_ is remarkably different, as 95 %-credible intervals barely overlap. This result can bear on the issue of whether infection transmission should depend on the density or the frequency of infectious individuals [32, 33]. In the model, we have assumed that the transmission probability per individual is constant. Alternatively, we could have adhered to the more usual assumption that transmission probability is inversely proportional to the number of individuals in that setting [34]; in the case of school transmission, we should have used *q*_*s*_(*A*)=*c*/*n*_*s*_(*A*) and *q*_*s*_(*B*)=*c*/*n*_*s*_(*B*). As *n*_*s*_(*A*)≈1.5*n*_*s*_(*B*), this results into *q*_*s*_(*B*)≈1.5*q*_*s*_(*A*). The mean estimated *q*_*s*_ for school B is about 3 times the estimated mean for school A, but 1.5 sits well inside the ratios of values in the 95 %-credible intervals. Thus we can conclude that a frequency-dependent transmission probability is fully compatible with our findings, whereas data are borderline with respect to rejecting density-dependent transmission.

The estimates of the within-school *R*_0_ (mean and 95 *%* CI: 0.85 (0.59-1.13) for school A, 1.09 (0.85-1.37) for school B) lie in the low end of the spectrum of values estimated from influenza spread in schools [35]. Similarly, the estimates of within-class and within-grade (but not those of within-school) transmission probabilities obtained in [14] are somewhat higher than ours. It is possible that such differences reflect actual behavioural differences between students in Pennsylvania and in Northern Italy. Alternatively, such differences may be simply due to stochastic variations in the school epidemics, which could also be the reason for the different estimates in schools A and B. In order to explore the plausibility of the latter explanation, in Section D of the Additional file 1 we show the median estimates of *R*_0_ obtained from different realizations of the stochastic model; although the parameter values are the same for all realizations and yield through (3) *R*_0_=1.48, the estimated *R*_0_ vary between 1.02 and 1.61 in 95 % of simulations. It has also to be remarked that many investigations have focused on schools where an unusually high infection spread had been observed [6] so that possibly the value of *R*_0_ estimated from them is higher than average. On the other hand, the two schools in our investigation have been chosen purely for convenience; thus, they may be more representative of usual behaviour of infection spread.

In particular, our model estimate of *R*_0_ lower than 1 for school A highlights the relevance of transmission from outside (with a likely crucial role of households) in maintaining the school outbreak, similarly to the findings of [14]. As information on household cases collected with the questionnaires was inadequate, we relied on two simple alternatives for transmission probability from outside school *ε*: either a constant, or proportional to the actual influenza incidence in the population. Concerning the latter, we could use only the weekly ILI incidence estimated through the surveillance system InfluNet [21] at the Trento province level, that, on the one hand, is much larger than the territory where the students of the two schools actually live, and, on the other hand, is smaller than the recommended aggregation level of sentinel data that makes them statistically significant; furthermore, the sentinel data may include information from the students in our population study, although their influence should be negligible given the structure of GP and surveillance systems. Despite these limitations, we deem that this choice yields the best available alternative to a constant probability of infection transmission from outside school. The outcomes of the comparison between the two model variants are not unequivocal: for school A model CGS with varying *ε* yields a larger DIC than model CGS with a constant *ε*, while for school B the model with varying *ε* performs much better than the model with constant *ε*. This statistical result reflects the different pattern in the distribution of cases (see Figure 1), but we cannot find any obvious explanation for it.

The value of *γ*=0.1 for the probability that the effective (at school) infectious period lasts 2 days has been extrapolated from limited data presented in [18], following the usual practice of estimating the generation time from household studies or other instances where dates of infections can be independently established [33, 36]. In Section D of the Additional file 1 Text, we show that a joint estimation of transmission probability and infectious period, as in the study by White et al. [37], is generally very difficult. Anyway, the main conclusions obtained on the differences between transmission probabilities in the different contexts and between two different schools do not depend on the exact value of *γ*; changing its value simply results in changing the numerical estimates of *q*_*c*_, *q*_*g*_ and *q*_*s*_ but not their relative features.

The model we used is very simplified in several respects, from employing a very stylized infectious period, to ignoring school closure during weekends or asymptomatic cases.

It has been estimated [38] that school closures during weekends contribute to decrease the effective reproduction number of about 8 *%*. Since the generation time in the school setting is short, weekends can break the transmission chain at school thus having an impact on the transmission pattern [27, 28, 34, 38]. On the other hand, household and outside transmission is likely to increase during weekends, as often assumed in modelling studies [34, 39]. Again, for the sake of simplicity, we preferred to avoid the introduction of parameters that may not be easily estimated, but in principle the model could be extended to distinguish between weekdays and weekends.

Our model assumes that all infections are symptomatic and lead to the same level of infectiousness. Indeed, using raw data, the estimate of children showing influenza symptoms is 52 *%*. This value is comparable to the estimate of 56.9 *%* infection rate for 2009 A/H1N1pdm09 in primary-school children in Italy, that was derived from serological data [40]; thus, it seems likely that only a small number of children in those schools got infected with influenza without showing symptoms. On the other hand, it is certainly possible that the fraction of children of the schools considered in our study that got infected was much higher than the national average of 56.9 *%*. Alternatively, it is possible that some of the children that reported symptoms were not actually infected with influenza virus, while others were infected but did not show symptoms. The lack of serological data prevents from a choice between different alternatives. Accordingly, we decided to use the most parsimonious alternative, namely to neglect asymptomatic infections.

Data from a questionnaire have several other shortcomings: a retrospective survey is prone to recall bias, and this may have affected the parameter estimates and the quality of the results. Furthermore, non-respondents may differ in several respects from those about which we collected information; for instance, it is possible that parents of students not infected or asymptomatically infected could have chosen not to respond to the questionnaire more often than parents of students with symptomatic infection. We tested the effect of some sources of errors in the analysis of simulated data (see Section D in the Additional file 1), assuming that, beyond missing data, 30 % of reported symptom dates were incorrect. Such errors seem not to bias the resulting estimates, but only to somewhat increase the width of credible intervals; however, other sources of errors (for instance, if no non-respondent had been infected, or vice versa) could lead to over- or under-estimation.

Despite these limitations, our analysis provides evidence of different influenza transmission in class and grade. We have shown that the MCMC algorithm used can yield plausible results even starting from incomplete and possibly inaccurate data (such as those derived from questionnaires); further and more detailed data (including serology as well) would be useful to improve the model and the corresponding estimates.

## Conclusions

The analysis of data on ILI during the 2009 H1N1 influenza pandemic collected through a questionnaire in two primary schools shows that within-class transmission has been higher (of a factor 3 to 4) than transmission to students in other classes, confirming the estimates obtained in [14] analyzing a school outbreak in Pennsylvania. The analysis thus confirms the relevance of targeting within-class transmission for controlling the spread of influenza in school settings, but also provides a quantitative estimate, useful for planning and assessing possible interventions. Since empirical evidence on within-school infection transmission is scarce, we believe the estimates obtained are quite relevant for the several mathematical models that include specific transmission routes through school contacts.

Several other results have emerged from the analysis:

Estimated average number of infected students per school case was close to 1, suggesting that household and community transmission played an important role in sustaining the school epidemics.

Transmission to students in the school (but in different classes) was lower in the larger school, confirming that number of contacts modulates the transmission strength.

## Additional files

Additional file 1 Supplementary material. (PDF 292 kb)

Additional file 2 Files of data on school A. Each row corresponds to a student for which we received the filled questionnaire. In the column ‘classe’ there is the name of the class (the 1st digit is the grade); in ’nstud’ the number of students in that class; in ‘infected’ whether that student showed ILI symptoms (1) or not (0); in ‘home’ the number of household members infected before that student (−1 if infected =0); ’day’ and ’month’ the date of ILI occurrence (−1 if infected =0). (TXT 4 kb)

Additional file 3 Files of data on school B. Explanations as for school A. (TXT 3 kb)
