# Supplementary material for: Estimating transmission probability in schools for the 2009 H1N1 influenza pandemic in Italy
Source: Theor Biol Med Model. 2016 Oct 12;13:19. doi: 10.1186/s12976-016-0045-2 (PMC5059896; doi:10.1186/s12976-016-0045-2)
Supplement: Supplementary file 1 — Supplementary material. (PDF 292 kb) [file 12976_2016_45_MOESM1_ESM.pdf]

Estimating transmission probability in schools for the 2009 H1N1 influenza pandemic in Italy

V. CLAMER[1], I. DORIGATTI[2], L. FUMANELLI[3], C. RIZZO[4], A. PUGLIESE[1]

[1] Department of Mathematics, University of Trento, Trento, Italy

[2] MRC Centre for Outbreak Analysis and Modelling, Department of Infectious Disease Epidemiology, Imperial College London, United Kingdom

[3] Center for Information Technology, Bruno Kessler Foundation, Trento, Italy

[4] Istituto Superiore di Sanità, Rome, Italy

Corresponding author: Andrea Pugliese, Via Sommarive 14, 38123 Povo - Trento (Italy) - andrea.pugliese@unitn.it

Supplementary Material

A Text of the questionnaire

The questionnaire was delivered to parents (or care-givers) of the schools' students in the first days of December 2009, and returned to school teachers. Its text was the following:

Has your child, student in the school XY, been sick of influenza-like syndrome (*) since October 15?
(*) It is defined as influenza-like syndrome:
fever $\geq 38°$C, together with at least one of the following symptoms:

- head-ache
- malaise
- shivering
- extreme fatigue

and at least one of the following respiratory symptoms:

- cough
- sore throat
- nasal congestion.

YES/NO.
If the answer is yes, please report the date of the first symptoms.
For each member of the same household, please report the age, whether it has been sick of influenza-like syndrome and, if so, the date of the first symptoms.

B The algorithm used

The available data are

1. whether students have shown influenza symptoms or not;
2. the day of symptom onset.

All this information will be named Z. For the moment, we neglect the problem of non-respondents.
We wish to obtain *a posteriori* distributions for the parameters q_s, q_A, q_B (q_A and q_B are functions of q_g and q_c, see below) and γ (collectively named ϑ). As computing the likelihood of the data Z would be very complex, we denote Y the dates of infection of the individuals with symptoms onset, and we choose

$$f_{prior}(\vartheta, Y) = P(Y|\vartheta)\pi(\vartheta)$$

where $\pi(\vartheta)$ is the chosen *a priori* (uniform) distribution for the real parameters ϑ.
Then, through Bayes' formula

$$f_{post}(\vartheta, Y) \propto P(Z|\vartheta, Y) f_{prior}(\vartheta, Y) = \frac{P(Z, Y|\vartheta)}{P(Y|\vartheta)} P(Y|\vartheta)\pi(\vartheta) = P(Z, Y|\vartheta)\pi(\vartheta). \qquad (1)$$

where

$$P(Z, Y|\vartheta) = \prod_{j,h} \prod_{t=t_{min}}^{T_{max}-1} \gamma^{I^{j,h}_{2_{t+1}}} (1-\gamma)^{(I^{j,h}_{1_t} - I^{j,h}_{2_{t+1}})} P(S^{j,h}_{t+1}|S^{j,h}_t, I^{j,h}_t, I^j_t, I_t). \qquad (2)$$

$P(S^{j,h}_{t+1}|S^{j,h}_t, I^{j,h}_t, I^j_t, I_t)$ can be obtained from equation (2) of the main text , and the quantities $S^{j,h}_t$, $I^{j,h}_t$, I^j_t, I_t can all be easily computed from Y and Z.
The transition probabilities $p^{j,h}_t$ used in eq. (2) of the main text can be written in a computationally more efficient way, by introducing the quantities q_A and q_B as

$$1 - q_A = \frac{1-q_c}{1-q_g}$$
$$1 - q_B = \frac{1-q_g}{1-q_s}$$

as

$$p^{j,h}_t = (1-q_A)^{I^{j,h}_t} (1-q_B)^{I^j_t} (1-q_s)^{I_t} (1-\varepsilon) \qquad (3)$$

using the quantities

$$I^j_t = \sum_{l=1}^{n_j} I^{jl}_t \quad \text{(total number of infectious in grade } j\text{)}$$
$$I_t = \sum_{m=1}^{5} \sum_{l=1}^{n_m} I^{ml}_t \quad \text{(total number of infectious in the school)}$$

Non-respomdents are handled by including in the vector of added parameters Y the state (eventually infected or not) and, if so, the dates of infection and removal for all students whose information is missing.
The posterior distribution is estimated as the stationary distribution of the Markov chain obtained implementing the Metropolis-Hastings algorithm [2, 4].

C Parameters updating

To update the parameters we use a Single Component Metropolis-Hastings (see [2]). Denote y the current parameter (either q_c or q_g or q_s or ε). We update the parameter through the formula $x = \frac{ye^{ra}}{1-y+ye^{ra}}$ where x denotes the proposed updated parameter, a is from a normal and r has been adjusted to obtain good mixing.

So, by simple calculations, we obtain that,

$$q(y|x) = \frac{1}{y(1-y)\sqrt{2\pi}r} e^{-\frac{1}{2r^2}[\log(x)+\log(1-y)-\log(y)+\log(1-x)]^2}$$

$$q(x|y) = \frac{1}{x(1-x)\sqrt{2\pi}r} e^{-\frac{1}{2r^2}[\log(x)+\log(1-y)-\log(y)+\log(1-x)]^2}$$

and then $\frac{q(y|x)}{q(x|y)}$ is equal to

$$\frac{q(y|x)}{q(x|y)} = \frac{x(1-x)}{y(1-y)}.$$

As the days of infection are discrete variables, we updated them by a different method. Precisely, every cycle we randomly selected a class, and 3 students with symptoms within that class, to be updated; since our assumptions leave only two choices for the infection day, we simply changed the differences (*infection day*) − (*symptoms day*) from −1 to −2 or from −2 to −1 for the 3 infected students.

We proceeded similarly for the infection status of missing data, and for their symptoms day, allowing, in the latter case, for all possible days of observation.

D Test on simulated data

D.1 Joint estimation of transmission probability and infectious period

We started with the simple case of a single class, aiming at the estimate of the probabilities to remain infectious for two days, γ, and to be infected from someone inside the same class, q_c. We performed a series of simulations varying γ from 0.1 (the probability to remain infectious for two days is very low) to $\gamma = 0.9$ (the probability to remain infectious for two days is very high) with a step of 0.1; correspondingly, q_c is changed in such a way that R_0 (in this case $nq_c(1+\gamma)$) remains constant.

In Table S1 the parameters values used to simulate the model are shown, both for a class of $n = 25$

Simulation set	γ	$n = 25$ q_c	$n = 250$ q_c
1	0.1	0.054	0.0054
2	0.2	0.049	0.0049
3	0.3	0.046	0.0046
4	0.4	0.042	0.0042
5	0.5	0.039	0.0039
6	0.6	0.037	0.0037
7	0.7	0.035	0.0035
8	0.8	0.033	0.0033
9	0.9	0.031	0.0031

Table S1: *Parameter values for the probability of remaining infective for two days γ, and the class infection probability q_c used in the 9 sets of simulations as to have $R_0 \approx 1.48$ with a class of $n = 25$ students and the fictional case of a class of $n = 250$ students.*

children and for a (fictional) case of a class of 250 children. Figure S1 shows the results obtained in the case of a class of $n = 25$ children or in the (fictional) case of a class of 250 children.

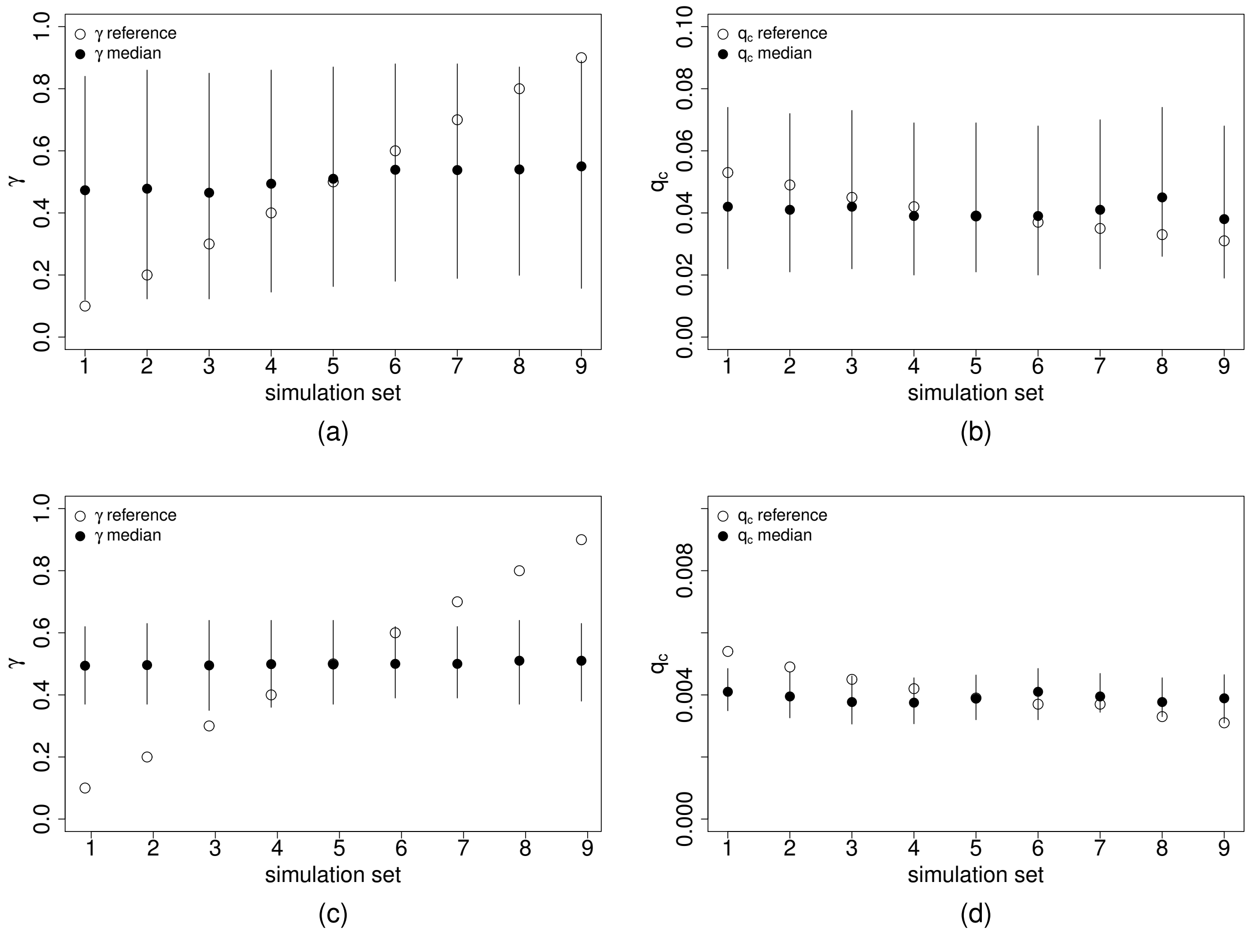

Figure S1: *Estimates of γ (panels a and c) and of q_c (panels b and d) in 9 sets of simulations performed by varying γ and q_c while keeping $R_0 \approx 1.48$ (in Table S1). Panels a) and b) have $n = 25$, c) and d) have $n = 250$. The reference values used in the simulations are represented as white dots, while the medians of the posterior distribution are represented as black dots, with the bars representing 95%-credible intervals.*

It may be noticed that the mean value of estimated γ is always close to 0.5, independently of the value of γ used in the simulation. Increasing n reduces the width of the credible intervals, but does not remove the bias. Also the mean estimated value of q_c is almost constant, independently of the value used in the simulations, at the value that would be correct for $\gamma = 0.5$.

Because of this problem, in all the following simulations we set γ equal to 0.1 without estimating it.

D.2 Simulations of school model

All the following simulations are based on the same school structure, similar to the one of school A: a total of 15 classes, 3 for each of the 5 different grades, and each class composed of 25 children (according

Simulation set	q_c	q_g	q_s
1	0.0037	0.0029	0.0037
2	0.0066	0.0037	0.0033
3	0.009	0.0042	0.003
4	0.011	0.0044	0.0028
5	0.013	0.0046	0.0026
6	0.015	0.0047	0.0024
7	0.016	0.0048	0.0023
8	0.018	0.0049	0.0022
9	0.019	0.00497	0.0021
10	0.02	0.005	0.002

Table S2: *Parameter values of the class infection probability q_c, the grade infection probability q_g and the school infection probability q_s used in the 10 sets of simulations.*

to the Italian primary schools structure).

We generated data under different parameter values, ranging from the case where q_c, q_g and q_s are approximately equal (transmission homogeneous among all students in a school) to another one where $q_c = 10q_s$ and q_g is intermediate (transmission is higher to students in the same class, then to those in the same grade, and lowest to all other students of the school). The parameter values have been chosen so as to have (using formula (3) of the main text) $R_0 \approx 1.48$ [1, 3, 5, 6].

Precisely, we performed 10 sets (labeled 1 to 10) of 50 simulations with the parameter values shown in Table S2; for each simulation we ran the MCMC algorithm to obtain posterior distributions of the parameters q_c, q_g, q_s and ε. The results are shown in panel a) of Figure S2.

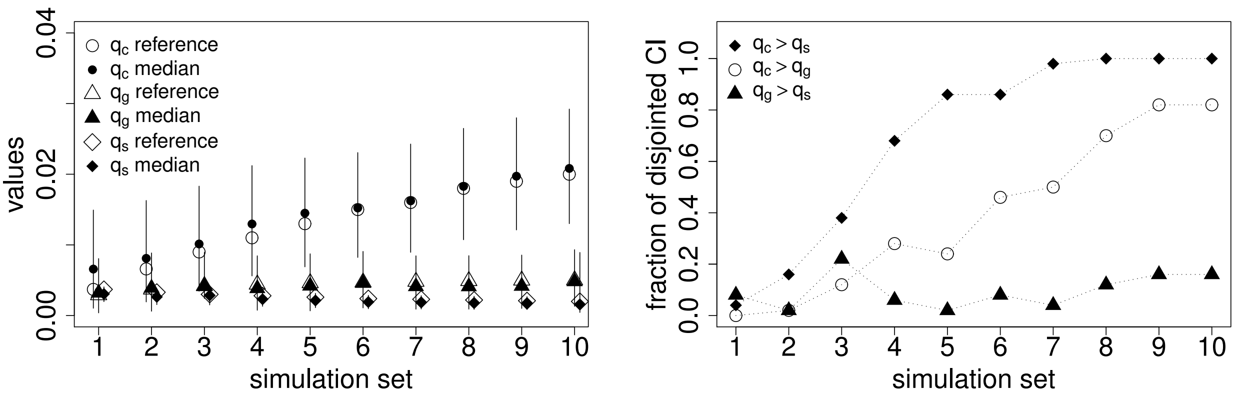

Figure S2: *(a) Ten sets of 50 simulations starting from reference values represented as white dots (in Table S2) and $\varepsilon = 10^{-3}$. The median estimated values are represented as black dots with the 95%-credible intervals (b) Fraction of simulations for which the 95%-credible intervals for the different parameters do not intersect.*

It can be seen that the estimates are reasonably correct, with the mean of the posterior distributions around the reference values. It may be noticed, however, that, when the three parameters q_c, q_g and q_s are close to each other, the algorithm tends to overestimate q_c, the transmission rate in the same class.

We also examined whether the 95%-credible intervals for the three parameters intersect each other. The answers are shown in Figure S2 (panel b)).
It can be seen that, when the parameters are indeed equal, around 5% of the times one obtains non-intersecting 95%-credible intervals, something close to expectations.
On the other hand, a difference is picked up almost always between q_c and q_s from set 4 onwards (i. e. when the ratio $q_c/q_s \approx 3.8$) and between q_c and q_g from set 8 onwards (i. e. when the ratio $q_c/q_g \approx 3.7$), while the ratio q_g/q_s never reaches such values, and thus a difference between q_g and q_s is seen only occasionally in the simulations.

Finally we present in Figure S3 the estimates obtained for R_0. In order to display the variability among simulations, we show for each simulation only the median estimate of R_0: in the left panel, this is obtained by using formula (3) of the main text on the joint posterior distribution of (q_c, q_g, q_s) computed with the MCMC algorithm as described above; in the right panel it is obtained using $R_0 = 1 + rT_I$ where

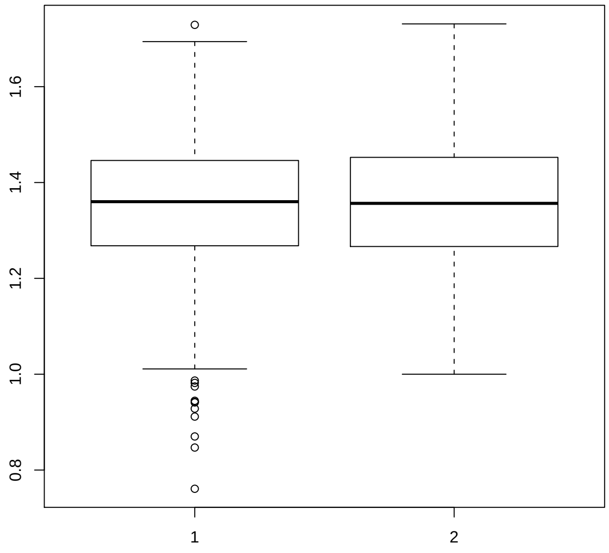

Figure S3: *Box plots of the median estimates of R_0 obtained in each simulation described in Figure S2: left, using formula (3) of the main text on the joint posterior distribution of (q_c, q_g, q_s); right, using $R_0 = 1 + rT_I$ with r estimated from the curve of cumulative cases. The bottom and top of the box are the first and third quartile of the distributions, so its width is the inter-quartile range (IQR); the line in between is the median; the whiskers extend to the most extreme estimates that are not farther away than 1.5 IQR from the quartiles; small circles represent further estimates.*

r is the median estimate obtained by fitting (on different time intervals) a linear model to the curve of cumulative cases (in logarithmic scale), as explained in the main text. With both methods, the median value of R_0 is 1.36 (against $R_0 = 1.48$ using formula (3) of the main text with the parameter values used in the simulations); the width of the boxes and of the whiskers give a visual impression of the variability in estimates among different simulations.

D.3 Test with missing data

To test the behavior of the algorithm when dealing with missing data, as in the case of actual data, we re-analyzed the previous simulations, but assuming that a random 20% of the data of each class is not reported and thus cannot be used in the estimation procedure. The results obtained are shown in Figure S4 (panels a) and b)). The results can be considered almost as satisfactory as in the case without missing data. As expected, the credible intervals are somewhat wider than in the case without missing data, and thus they intersect somewhat more often. It can also be remarked that, with missing data, the transmission rate inside the class q_c is on average overestimated in all simulation sets.

D.4 Test with missing data and errors

We further tested the algorithm by assuming that 20% of the simulated data in each class are not reported and that only 70% of the reported dates are correct, while 20% of them are shifted of ± 1 day and the remaining 10% of ± 2 days. Figure S4 (panels c) and d)) shows the estimates obtained in this scenario. Also in this case the estimation of the data is reasonably correct and this indicated that we have obtained a robust result, although the absolute value of q_c is somewhat overestimated when the parameters are close to each other.

References

[1] **Ghani AC, *et al.*** The Early Transmission Dynamics of H1N1pdm Influenza in the United Kingdom. *PLoS Currents* 2009; **1**: RRN1 130.

[2] **Gilks WR, Richardoson S, Spiegelhalter DJ.** *Markov Chain Monte Carlo in practice.* Chapman & Hall/CRC, London, 1996.

[3] **Yang Y, *et al.*** The transmissibility and control of pandemic influenza A(H1N1) virus. *Science* 2009; **326**: 729-733.

[4] **O'Neill PD.** A tutorial introduction to Bayesian inference for stochastic epidemic models using Markov chain Monte Carlo methods. *Mathematical Biosciences* 2002; **180**: 103-114.

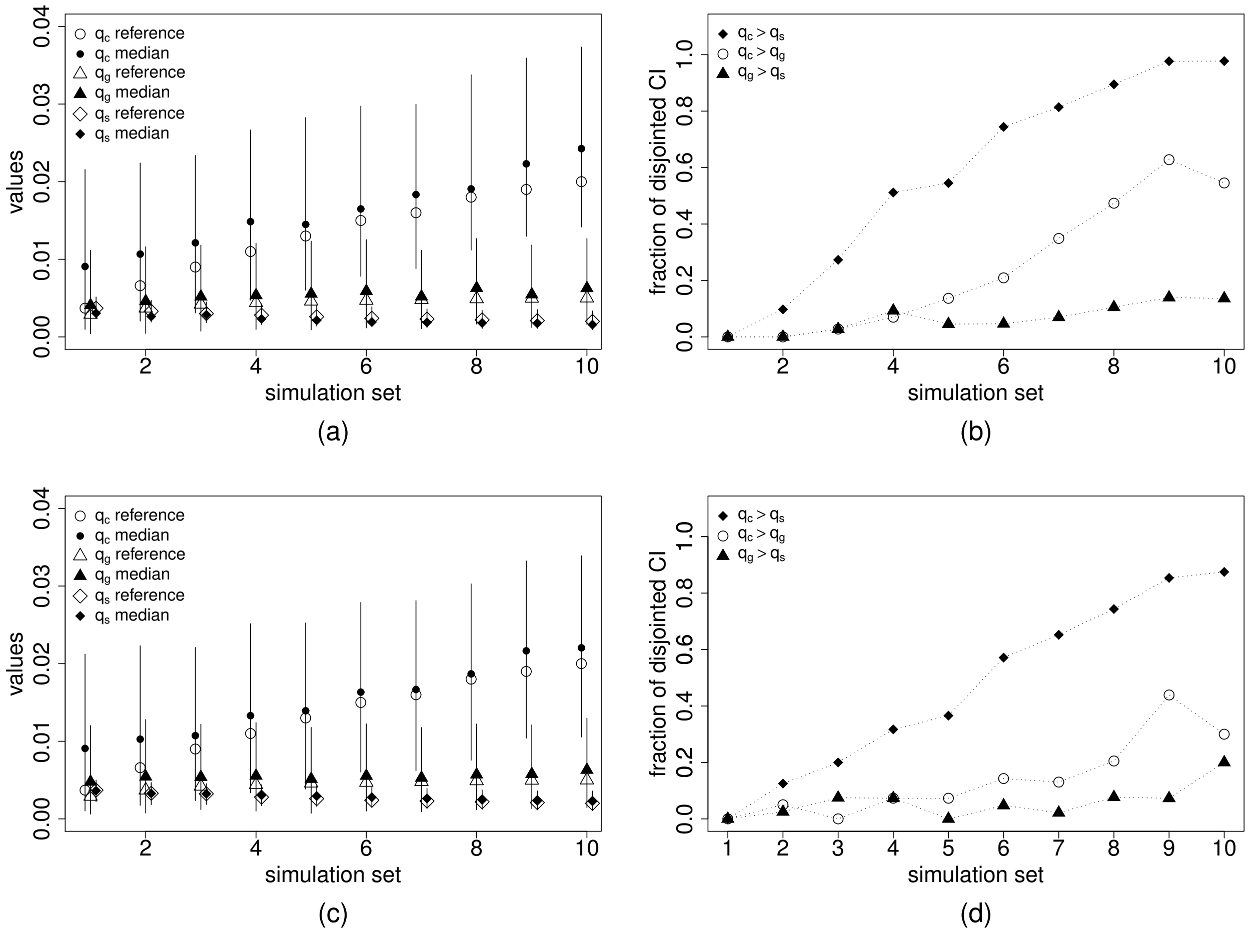

Figure S4: *Fifty simulations (panels a and c) starting from reference values represented by white dots (in Table S2) and* $\varepsilon = 10^{-3}$ *by assuming that* 20% *of the data of each class are not reported. The median estimated values are represented as black dots with the* 95%*-credible intervals. Fraction of simulations in which the* 95%*-credible intervals for the different parameters do not intersect in panels b) and d). In panels a) and b)* 20% *of the data are considered to be missing. Beyond that, in panels c) and d)* 70% *of the reported dates are correct,* 20% *are shifted of* ±1 *day and the remaining* 10% *of* ±2 *days.*

[5] **Fraser C, *et al.*** Pandemic potential of a strain of influenza A (H1N1): early findings. *Science* 2009; **324**: 1557-1561.

[6] **Pourbohloul B, *et al.*** Initial human transmission dynamics of the pandemic (H1N1) 2009 virus in North America. *Influenza and Other Respiratory Viruses* 2009; **3**: 215-222.
